# Bullying against Healthcare Professionals and Coping Strategies: A Scoping Review

**DOI:** 10.3390/ijerph21040459

**Published:** 2024-04-09

**Authors:** Ana Rita Valente Ribeiro, Ana Isabel Sani

**Affiliations:** 1 Faculty of Human and Social Sciences, University Fernando Pessoa (UFP), 4249-004 Porto, Portugal; rita.valente10@gmail.com; 2 Fernando Pessoa Research, Innovation and Development Institute (FP-I3ID), Observatory Permanent Violence and Crime (OPVC), University Fernando Pessoa (UFP), 4249-004 Porto, Portugal; 3 Research Centre on Child Studies (CIEC), University of Minho, 4710-057 Braga, Portugal

**Keywords:** workplace bullying, coping strategies, healthcare professionals, Negative Acts Questionnaire—Revised (NAQ-R)

## Abstract

Violence against healthcare professionals is an event that further burdens the daily lives of those who try every day to care for and assist those who need it most. In an attempt to overcome these events, there are coping strategies that can be used to reduce the stress caused. Therefore, this study aims to analyse the phenomenon of violence against healthcare professionals and the relationship between the bullying suffered by these professionals and the coping strategies they developed to overcome these moments. To this end, a scoping review was conducted in which eight articles were selected for final analysis from a total of 276 articles found in three electronic databases (EBSCO, PubMed, and Web of Science). This review concludes that the most common workplace bullying behaviours include excessive workloads, humiliation and ridicule, impossible deadlines, and verbal attacks. Professionals reported negative impacts, such as helplessness, depression, stress, insomnia, and the desire to change jobs. Victims of workplace bullying often expressed their intention to leave their current job or even abandon the profession. Problem-focused coping strategies are the most used. The studies indicated that workplace bullying negatively affects professionals in physical and mental terms, as well as in terms of quality of life at work, requiring more research and adoption of preventive measures to identify and combat the problem.

## 1. Introduction

Bullying understood as aggressive behaviour, intentional and repeated over time, by one or more people towards someone with the purpose of causing harm [1] is not a behaviour exclusive to the school environment and carried out only at children and young people. Bullying is a conduct that a person, of any age group, gender, or profession, may adopt in various other contexts, such as the workplace [2].

The definition of bullying in the workplace is multifaceted and complex, with several definitions and terminologies that different authors have adopted to describe this phenomenon. In the United States of America, Brodsky [3], an American psychiatrist, began to use the expression harassment, which he defined as repeated and persistent behaviour with the intention of frustrating or receiving some type of reaction from the victim. On the other hand, Leymann [4,5], a Swedish researcher, began to use the expression mobbing, as he stated that bullying was more physical and threatening violence and then defined mobbing as a more sophisticated behaviour, giving as an example isolation of the victim. Hirigoyen [6], a French psychiatrist, uses the expression “moral harassment” where she considers that behaviours can affect a person’s dignity and integrity and even put their job in danger. The term incivility, although less frequent in articles that address the topic, is sometimes used to refer to acts of rudeness and discourtesy, rumours and gossip, and disrespect for the dignity of the professional, including by colleagues [7]. The literature states that these and other actions that compromise a safety culture are extremely dangerous for both patient outcomes and the general well-being of healthcare personnel [8].

Although there are several definitions of bullying in the workplace, there are some similarities between all the definitions. In general, ‘workplace bullying’ can be defined as a type of violence involving recurrent practices through aggressive behaviours that occur over a period of time, which are undesirable by the victim [9,10]. Bullying in the workplace is a reality in many countries [11,12,13,14], particularly affecting professionals in the health sector [15,16,17,18]. 

Bullying in the workplace can lead to the emergence of physical and psychological problems, such as anxiety, depression, stress, and risk of death [19]. For some healthcare professionals, such changes may interfere with their ability to perform their work, such as providing care and referrals to health care. These experiences can be a factor of great stress in the lives of healthcare professionals, leading to serious consequences not only for professionals, but also for users and organizations [20]. To this end, there are some strategies that are studied as mediating variables (e.g., intention to quit, job engagement, job satisfaction) or moderators (e.g., social support, constructive leadership, attribution style) to face situations of harm, threat, and challenge to which are confronted [21].

The coping process is perceived as a set of efforts made by individuals when faced with some situations that may cause threat or discomfort to the person [22]. Coping strategies can be divided into two types, emotion-focused and problem-focused [23]. In emotion-focused coping strategies, energy is directed at somatic and/or feeling levels and aims to reduce the uncomfortable physical sensation caused by the state of stress. On the other hand, in problem-focused coping strategies, the victim tries to understand and know which problem is causing stress and acts directly to resolve the problem to try to change or avoid it in the future [23]. Authors like Rodriguez et al. [24] studied the differences in coping strategies between genders, concluding that men tend to use problem-focused strategies more, while women prefer to use emotion-focused strategies more.

Coping strategies are related to contextual factors. The individual may choose to adjust their strategy based on the specific situation that is causing the stress. It is important to note that there are no adaptive or maladaptive coping strategies [25]. The decision on which strategy to adopt is a personal choice, the individual chooses the strategy that they consider positive and that helps to alleviate discomfort and reduce negative feelings associated with stress. In contrast, coping strategies are considered negative and ineffective if they are applied, but the stressful situation continues to persist and maintains an imbalance in several aspects [25,26].

Folkman and Lazarus [27] created a transactional model of coping where they argue that stress is contextual, representing a process of interaction in constant evolution between the individual and the environment. In this context, stress is defined as a situation that the individual evaluates as significant and that imposes needs that exceed the resources available to deal with the moment. This model comprises four essential elements: (1) the conception of coping as a process that occurs between the individual and the environment; (2) its main purpose being to deal with stressful situations; (3) the involvement of assessment, that is, how the person perceives stress; (4) the implication of the mobilization of resources, in which individuals use cognitive and behavioural resources to deal with claims that arise both from within and outside the interaction with the environment [22,27].

According to Angst [28], the term resilience is directly related to coping strategies, as it can be considered as a procedural form between the individual and the environment that surrounds them, and the latter must evaluate and interpret the phenomena they perceive. Thus, people who use coping strategies can be considered resilient people.

Therefore, this scoping review aimed to explore the phenomenon of bullying suffered by healthcare professionals and the coping strategies used to combat it. A review would be important to explore the phenomenon of bullying suffered by healthcare professionals and the coping strategies used, as this is a significant and harmful problem that affects the mental health and well-being of healthcare professionals. Bullying can have profound impacts on the quality of work, team morale, and patient safety. This scoping review can contribute to raising awareness about this issue, promoting changes in organizational practices, and providing support to healthcare professionals facing this form of violence in the workplace. Understanding the coping strategies used by healthcare professionals can help identify which ones are most effective and develop interventions to support those experiencing bullying. This scoping review provides a comprehensive overview of existing research on this topic, allowing for the delineation of studies in this area. 

Thus, this scoping review is presented in an operationalizable and replicable manner. Its goal is to encompass a specific body of literature on the phenomenon of violence against healthcare professionals and the relationship between the bullying suffered and the coping strategies used to face these most stressful moments, examine its research trends, and potentially pave the way for a systematic review to pinpoint research gaps and provide recommendations for future studies. Therefore, we sought to identify the types of bullying behaviours that affect healthcare professionals and examine the possible consequences of these behaviours in terms of physical and mental health. Such as investigating the coping strategies that healthcare professionals adopt to deal with bullying in the workplace. To this end, the following questions arise:

1. What are the main bullying behaviours identified by healthcare professionals and what are their consequences?
2. What coping strategies are used by healthcare professionals who are victims of bullying?

## 2. Methodology

### 2.1. Research Procedures

For this scoping review, a search was carried out in the following databases: EBSCO, Web of Science, and PubMed. To this end, the following combinations of keywords were used: (Profissionais de saúde OR healthcare professionals OR médicos OR doctors OR physicians OR nurses OR enfermeiros) AND (Bullying OR mobbing OR *civility) AND (Estratégia* de coping OR coping strategie* OR cope OR coping OR coping skill*) OR (NAQ-R OR Negative Acts Questionnaire-Revised). To obtain the most relevant results from all databases, some restrictions were used. In the EBSCO database, the following restrictions were used: “Full Text”, “Peer Reviewed”, “Available to library collection”. In Web of Science and PubMed, they were restricted to “Open Access” and “Full Text”, respectively. On all platforms, the language was restricted to Portuguese, English, and Spanish, and the search was restricted to article dates greater than the year 2010 (Supplementary Materials). The research was carried out in May 2023.

### 2.2. Inclusion and Exclusion Criteria

In order to guide the selection of studies, some criteria were defined:

Inclusion criteria: (a) studies that addressed violence against healthcare professionals and coping strategies; (b) studies in which the population was doctors and/or nurses; (c) studies in Portuguese, English, and Spanish; (d) publications dated after 2010.

Exclusion criteria: (a) studies with a student population; (b) scoping reviews, systematic reviews, or meta-analyses; (c) studies carried out with professionals other than doctors and/or nurses; (d) studies that covered only one of the variables under study; (e) publications written in a language other than Portuguese, English, and Spanish; (f) publications dated earlier than 2010 (a report on the Assessment of Episodes of Violence Against Health Professionals was released by the General Directorate of Health [29]). By focusing on a period after 2010, we can ensure that the data and findings discussed in the report are still accurate and reflective of the current situation regarding violence against health professionals. Additionally, as the review was carried out in May 2023, it is important to consider more recent information to provide the most current and reliable analysis and recommendations.

### 2.3. Article Selection

**Figure 1:**
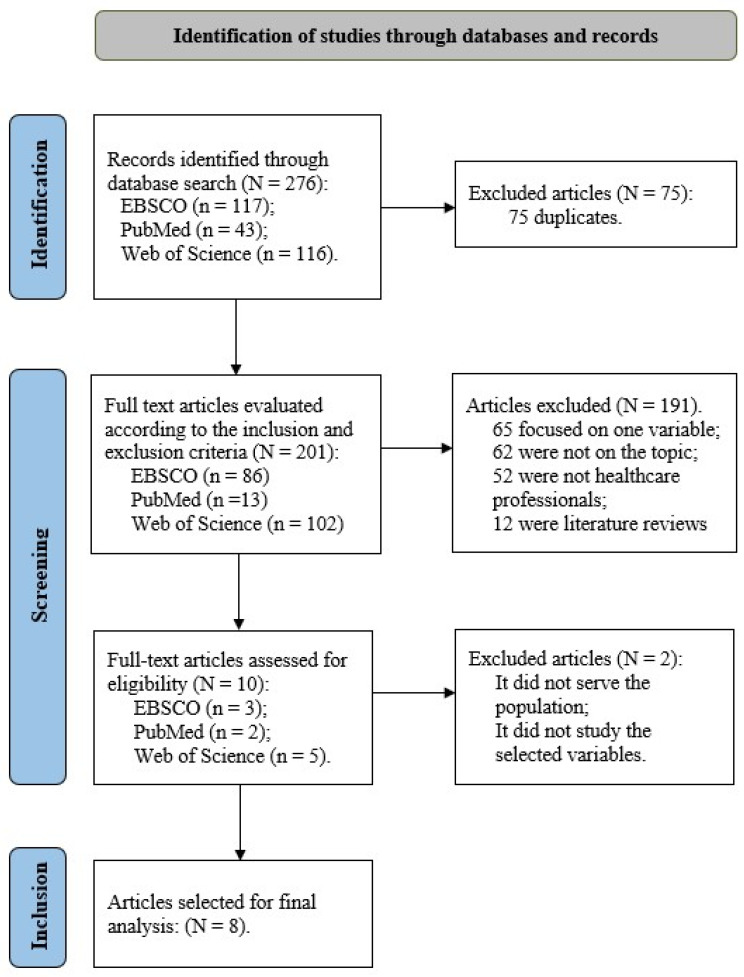
Article selection flowchart (retrieved from http://www.prisma-statement.org/, accessed on 20 march 2024).

The study followed the guidelines for scoping review [30] and was conducted in multiple stages involving article selection based on predefined criteria [31]. The project was registered [DOI 10.17605/OSF.IO/H7TAS]. The process ultimately yielded a final set of articles for analysis (cf. Figure 1) [32].

Initially, a search across three electronic databases yielded a total of 276 articles (117 from EBSCO, 43 from PubMed, and 116 from Web of Science). After excluding 75 duplicate articles, the remaining 201 articles underwent further screening.

During the second stage, articles were reviewed based on title and abstract to exclude those not relevant to the dissertation topic. This step resulted in the exclusion of 191 articles (86 from EBSCO, 13 from PubMed, and 102 from Web of Science): 65 articles focused on only one variable, 62 were unrelated to the intended topic, 52 did not study the intended sample, and 12 were literature reviews. 

In the final stage, the ten selected articles were thoroughly reviewed to identify their relevance to the study objectives. As a result, eight articles were deemed suitable for inclusion, while two were excluded for not meeting the defined criteria—one did not study the intended sample, and the other focused on only one of the variables of interest in this study.

### 2.4. Article Quality Analysis

To carry out the analysis of the quality of the articles, a checklist [32] was used in which 13 evidence-based items (Table 1) were evaluated to assess the solidity of the articles. In this checklist, six important points are evaluated: (I) the clarity of the title; (II) the structure of the summary; (III) the state of the art and the objectives of the study; (IV) the study protocol, eligibility criteria, sources of information, inclusion/exclusion criteria, information collection and study design; (V) summary of the main results; (VI) limitations found in the study.

In the checklist, articles are classified using a Likert scale in which the score varies between 0 and 2. A score of 0 indicates that the study lacks information on the topic at hand, whilst a score of 1 indicates that the researchers briefly acknowledged the problem without offering thorough explanations. A score of 2 indicates that the researchers extensively examined the issue and presented their technique in a clear and understandable way. Two researchers independently examined each article using the same grid. Items with different scores were assessed together to achieve the final values shown in Table 1. The researchers agreed by 95%. Table 1 shows the eight articles selected for this scoping review.

## 3. Results

The articles analysed all highlighted the negative impact of workplace violence on healthcare professionals in both their professional and personal lives. To conduct a more focused analysis, key data were extracted from each article, considering the fact that they were all based on empirical studies. Table 2 includes information on the authors and publication year, goals, countries where the studies were conducted, sample size, research instruments, design, and main findings related to the research questions. The full reading of the screened articles facilitated an exhaustive comparative analysis (horizontal and vertical) of the aspects studied, leading to an integrated cross-sectional analysis and extraction of the main results to answer the initial research questions.

### 3.1. Multiple Approaches to Workplace Bullying Research

Except for the study by Berry et al. [33], which is mixed in nature (i.e., both qualitative and quantitative), all of the other investigations were quantitative in nature. Some studies had more specific goals, like examining the effects of bullying in the workplace and various symptoms of post-traumatic stress disorder caused by bullying, as well as anxiety states and the coping strategies used by nurses [33,35]. These selected empirical articles were based on objectives as diverse as determining the type and frequency of negative behaviours in the workplace as well as the coping strategies used when exposed to such behaviours [34,40]. In a different study that was examined, the authors aimed to determine whether bullying victims’ coping mechanisms were less adaptive to stressful situations than those of nurses who were not bullied and whether bullying and anxiety are mediated by coping mechanisms [38]. A few of the chosen studies sought to understand the mediating function of resilience in a study on workplace bullying and professionals’ quality of life, or they sought to ascertain the relationship between workplace bullying and nurses’ quality of life [37,39]. Another study that was chosen examined the possibility that nurses’ leadership and humour orientation styles could affect how they perceive bullying in the workplace [36].

Overall, the studies emphasize the multifaceted nature of workplace bullying research and the importance of considering various perspectives and factors in addressing this prevalent issue. The inclusion of both qualitative and quantitative approaches, as seen in the study by Berry et al. [33] allows for a more holistic understanding of the issue.

### 3.2. Methodological Aspects of International Research on Workplace Bullying

The studies provide a glimpse into various aspects of nursing practice and research from different countries, including Australia [34,39], Korea [35,40], the United States of America [33,36], Norway [38], and China [37]. Each study had specific inclusion criteria for participants, ranging from newly licensed nurses to experienced ones, working in different healthcare settings (e.g., clinicals, hospitals). The methods used for data collection also varied, from disseminating questionnaires in contexts to utilizing online platforms like Survey Monkey [36,39] and Google Forms [35]. Some studies [33,38] stood out for involving two phases of investigation, showcasing a longitudinal approach to understanding nursing practices. The recruitment methods also differed, from contacting nurses through professional organizations to utilizing social media platforms like WeChat [37]. 

In this review and for the development of empirical studies on the topic, it was particularly important to know the diversity of assessment instruments used and their scope.

Hawkins et al. [34] used two quantitative instruments and a group of questions with specific objectives. One of the instruments was the Negative Acts Questionnaire—Revised (NAQ-R) [41], which aims to measure exposure to bullying in the workplace, consisting of 22 items that are divided into three subscales. The other instrument was the Ways of Coping Questionnaire [23], designed to examine an individual’s coping strategies in stressful situations, consisting of 66 items from eight domains of three types. The group of questions was developed from the literature and consisted of nine items.

In the study by Yoo and Ahn [40], three quantitative instruments were used: the Workplace Bullying in Nursing—Type Inventory (WPBN-TI) [42], developed to measure experiences of bullying in the workplace and consisting of 16 items; the Workplace Bullying in Nursing—Consequence Inventory (WPBN-CI) [43], built to measure responses to bullying in the workplace and consisting of 13 items; and finally, the Way of Coping by Lazarus and Folkman [23], revised and translated into Korean by [44], used to measure coping strategies and consisting of 33 items.

Mills et al. [36] used three quantitative instruments to achieve the objective of the study: the Multidimensional Sense of Humour Scale (MSHS) [45], consisting of 24 items and used to assess the sense of humour; Bass’s Multifactor Leadership Questionnaire (MLQ) [46], a measuring instrument consisting of five factors to identify three leadership styles; and finally, the Negative Acts Questionnaire—Revised (NAQ-R) [41].

In the study by Hong et al. [35], the authors used three quantitative instruments: the Workplace Bullying in Nursing—Type Inventory (WPBN-TI) [42]; the Korean version of the Impact of Event Scale—Revised by Lim et al. [47], consisting of 22 scales divided into three subscales, used to assess nurses’ post-traumatic stress; the Korean version of the Ways of Coping Checklist (WCCL) by Park and Lee [48], modified from the original version by Folkman and Lazarus [23], consisting of 39 items divided into two subscales that aim to evaluate the coping strategies used by nurses.

Reknes et al. [38] used three quantitative instruments: the Negative Acts Questionnaire—Revised (NAQ-R) [41]; the Hospital Anxiety and Depression Scale (HADS-A) [49], used to measure the anxiety symptoms and consisting of seven items; and the reduced version of the Utrecht Coping List (UCL) [50], consisting of 22 items divided into two subscales, which measure the frequency with which respondents act in a specific way when faced with problems or unpleasant situations, defining seven coping styles.

The study by Peng et al. [37] used a total of three quantitative instruments: the Negative Acts Questionnaire—Revised (NAQ-R) [41]; the Chinese version of the 10-item Connor–Davidson Resilience (CD-RISC-10) [51], consisting of 10 items and used to assess psychological resilience; and the Chinese version of Professional Quality-of-Life Scale (ProQOL-CN) by Shen et al. [52], consisting of 25 items divided into three subscales.

Tabakakis et al. [39] used three quantitative instruments: the 10-item Conner–Davidson Resilience (CD-RISC-10) [51]; the Negative Acts Questionnaire—Revised (NAQ-R) [53]; and finally, the Practice Environment Scale of the Nursing Work Index (PES-NWI) [54], consisting of 31 items divided into five subscales and used to evaluate the work environment.

Regarding the study by Berry et al. [33], four quantitative instruments and interviews were used. The four instruments used were the Negative Acts Questionnaire—Revised (NAQ-R) [41], the 10-item Perceived Stress Scale [55], which aims to assess perceived stress in the last month, the 20-item subscale of the State Trait Anxiety Inventory [56] that assesses how participants feel at the time of the survey, and finally, the Post-traumatic Stress Disorder Checklist—Civilian Version (PCL-C) [57], which tracks post-traumatic stress symptoms and contains seventeen assessment items. In relation to the second part of the study, telephone interviews were carried out with a semi-structured script which addressed the behaviours suffered from bullying, what they did to deal with or prevent these behaviours, and other actions taken to continue working in the same hospital unit.

Overall, the diversity in research environments, participant criteria, data collection methods, and recruitment strategies in the studies reviewed highlights the broad scope and depth of healthcare professionals practice and research around the world.

### 3.3. Bullying Behaviours, Consequences, and Coping Strategies 

Some articles in this review mentioned the main workplace bullying behaviours that their participants were exposed to. In studies by Hawkins et al. [34] and Berry et al. [33], nurses who were victims of bullying expressed that the most common type of behaviour was work-related bullying, that is, exposure to excessive workloads, being humiliated or ridiculed in relation to their work, and receiving tasks with delivery of impossible deadlines, among others. The healthcare professionals in the study by Hong et al. [35] also mentioned that they received verbal attacks and inadequate work instructions.

Some authors also mentioned the negative impacts that healthcare professionals had after being exposed to various acts of bullying in the workplace. For example, in the study by Yoo and Ahn [40], nurses complained of helplessness, depression, stress, insomnia, and physical discomfort, and even complained that they made more mistakes in their work and that they wanted to change jobs. They also reported that long-term exposure to bullying behaviours significantly reduces nurses’ coping resources [37].

More than 50% of the participants in three of the articles studied reported that they already had the intention of leaving their current job, changing units, or even giving up the nursing profession, since the bullying behaviours to which they were exposed were a very important factor for dissatisfaction with current employment, which is a major consequence of workplace violence [34,39,40].

These results demonstrate that the bullying suffered by these healthcare professionals can have negative effects in physical and mental terms and also interfere with the quality of the service they provide and the quality of personal life.

Studies have shown that nurses used different coping strategies, both active and passive, to deal with the most stressful moments of bullying in the workplace. Some of the strategies used were, for example, turning to a family member or friend for advice, focusing on what you had to do next, finding a “friend to work with”, avoiding anyone who exposes you to these behaviours, and using music and prayer as a form of distraction, among others [33,34].

Studies that identify which strategies are used by healthcare professionals indicate that, according to the answers given in surveys, problem-focused coping strategies are the most used in this profession, such as focusing on what had to be done next, trying to analyse the problem to better understand it, and focusing on work to distract your mind [33,34].

## 4. Discussion

The main bullying behaviours identified by healthcare professionals in the workplace include personal attacks, such as insults, humiliation, and ridicule, as well as work-related behaviours like excessive workloads and tasks with impossible deadlines. Understanding workplace bullying behaviours typically involves retrospective self-report questionnaires, where participants recall and report acts, they may have experienced. The choice of assessment instruments depends on the specific goals of each study and the cultural context in which it is conducted. Researchers utilize various instruments to measure exposure to bullying, coping mechanisms, psychological effects, and organizational factors. This diversity in assessment tools contributes to a comprehensive understanding of the incidence, consequences, and associations of workplace bullying in healthcare settings. To explore the temporal relationships between exposure to workplace bullying, coping strategies, and outcomes such as psychological well-being among healthcare workers, mixed-method studies with a longitudinal design are important [33,38]. Such studies can provide insights into how experiences of bullying evolve over time and their impact on individuals’ well-being.

Upon reviewing the various themes and investigations within the scope of this review, it becomes evident that bullying constitutes a detrimental act with far-reaching consequences for healthcare professionals, spanning physical, mental, professional, and quality-of-life domains [19,20,58]. Individuals subjected to frequent or daily bullying at work often experience heightened levels of stress, anxiety, helplessness, depression, and post-traumatic symptoms [33,34,35]. Furthermore, healthcare professionals may lose their motivation to work in such environments, leading to the desire to change jobs or even leave the profession altogether [37]. Notably, workplace violence detrimentally impacts job satisfaction, ultimately affecting clinical decision making and performance [34,39,40], particularly among nurses who often bear the brunt of workplace bullying [7].

Identifying high-risk subgroups among healthcare professionals susceptible to workplace bullying underscores the need for targeted interventions aimed at mitigating violence and its repercussions [35]. The findings hold crucial implications for specific professional groups, such as nurses, necessitating a comprehensive understanding of prevalent bullying behaviours, including excessive workloads and verbal attacks, to enable effective recognition and resolution [7]. Given the adverse effects of bullying, such as helplessness, stress, and attrition from the profession, urgent interventions are warranted to support and safeguard healthcare workers [19].

Resilience emerges as a critical mediator in the association between workplace bullying and professional quality of life [37,39], highlighting the significance of resilience-focused programs for healthcare professionals, particularly early in their careers. These initiatives should address structural empowerment’s impact on professional satisfaction and burnout, while also enhancing interpersonal interactions and addressing vulnerabilities (primary prevention). Additionally, interventions aimed at mitigating the adverse effects of workplace violence (secondary or tertiary prevention) must be implemented promptly and continuously to curb the phenomenon [19]. Proposed interventions may include skills training, cognitive-based problem-solving programs, and education on workplace violence [59,60,61]. Providing support for victims and implementing beneficial activities to alleviate perceived negative repercussions are equally essential components of intervention efforts.

Moreover, the role of leadership styles and organizational culture is pivotal in preventing bullying within the healthcare context. Cultivating supportive and respectful work environments, where leaders engage with staff, employ positive interaction methods, and establish long-term goals, serves as a deterrent to workplace bullying [36]. For healthcare organizations and management teams, these findings underscore the imperative of fostering a conducive workplace environment to prevent bullying and safeguard the well-being of healthcare professionals. By understanding the coping strategies employed by healthcare professionals, organizations can furnish appropriate resources and support to help employees effectively manage workplace bullying [7]. Employers should assess the type and frequency of bullying incidents using empirical measures (see Section 3.2) and develop strategies to manage and reduce stress, combat employee fatigue, and bolster coping mechanisms associated with exhaustion, should workplace bullying be identified.

Despite the prevalence of bullying experienced by healthcare professionals, many employ coping strategies such as problem-focused mechanisms and seeking social support to mitigate its impact on their lives and professions [34]. However, studies indicate that bullied healthcare professionals exhibit lower frequencies of goal-oriented active coping strategies, which are effective only under minimal exposure to bullying behaviours [38].

Nevertheless, this scoping review has its limitations. Despite thorough searches, some relevant studies may have been missed due to unavailability or inaccessibility. Additionally, studies with nonsignificant findings might not have been included, potentially biasing the review. Nonetheless, this review contributes to understanding bullying against healthcare professionals and their coping strategies.

Future research should concentrate on developing and evaluating interventions to prevent and address workplace bullying in healthcare settings. Longitudinal studies can offer insights into intervention effectiveness over time and identify best practices for supporting healthcare professionals. Moreover, investigating the long-term effects of workplace bullying on the physical and mental health of healthcare professionals, as well as patient care quality, is essential. Exploring the effectiveness of training programs, support systems, and organizational policies can foster a positive work environment, enhance satisfaction and retention, and reduce instances of bullying. Addressing these issues will create safer, more supportive environments for healthcare professionals and ultimately improve patient care.

## 5. Conclusions

In conclusion, workplace bullying in healthcare settings poses a significant threat to the well-being and professional quality of life of healthcare professionals. The detrimental effects of bullying, such as stress, anxiety, depression, and attrition from the profession, highlight the urgent need for targeted interventions to support and safeguard healthcare workers. Resilience-focused programs, interventions to mitigate workplace violence, and support for victims are crucial components of efforts to address workplace bullying. Leadership styles and organizational culture play pivotal roles in preventing bullying, emphasizing the importance of cultivating supportive work environments. By understanding coping strategies and providing appropriate resources and support, healthcare organizations can effectively manage and reduce the impact of workplace bullying on healthcare professionals. Further investigation into workplace violence, particularly in sensitive sectors like healthcare, public safety, and education, is imperative due to its personal and societal ramifications. Collaboration among researchers, policymakers, and healthcare organizations is essential for translating research findings into actionable interventions that benefit healthcare professionals and enhance patient care.

## Figures and Tables

**Table 1:** Quality table of articles under analysis.

Articles/Topics	1	2	3	4	5	6	7	8	9	10	11	12	13	Total
Berry et al. [33]	1	1	1	1	2	2	2	1	2	2	2	1	1	19 Average
Hawkins et al. [34]	2	2	2	2	2	2	2	2	2	2	2	1	2	25 High
Hong et al. [35]	2	2	2	2	2	2	2	2	2	2	2	2	2	26 High
Mills et al. [36]	1	2	2	2	2	1	2	2	2	2	2	2	0	22 High
Peng et al. [37]	1	2	2	2	2	2	2	2	2	2	2	2	1	24 High
Reknes et al. [38]	1	1	2	2	2	2	2	1	2	2	2	2	2	23 High
Tabakakis et al. [39]	1	2	2	1	2	2	2	1	2	2	2	2	1	22 High
Yoo & Ahn [40]	1	1	1	1	2	2	2	2	2	2	2	2	0	20 Average

Topics analysed: 1—title; 2—abstract; 3—rationale; 4—objectives; 5—protocol; 6—eligibility criteria; 7—information sources; 8—inclusion/exclusion criteria; 9—data collection process; 10—study design; 11—main measures; 12—summary of main results; 13—conclusion and study limitations. Grades: 0—not reported/not specified; 1—unclear/reported to a certain extent; 2—adequately done.

**Table 2:** Summary of selected articles.

Authors (Year)	Goals	Local/ Participants	Instruments	Design	Main Results [Bullying, Symptoms, and Coping Strategies]
Berry et al. [33]	Determine differences in the perception of stress, state anxiety, and post-traumatic stress symptoms based on levels of exposure to bullying, as well as determine the strategies used	USA 1st phase = 84 nurses 2nd phase = 11 nurses	Negative Acts Questionnaire; 10-Item Perceived Stress Scale; 20-Item Subscale of the State Trait Anxiety Inventory; Post-traumatic Stress Disorder Checklist Civilian Version; Individual Questionnaire	Quantitative and qualitative study	The study identified significant differences in the perception of stress, anxiety, and post-traumatic symptoms that have been reported by people with frequent or daily exposure to workplace bullying behaviour.
Hawkins et al. [34]	Understand the type and frequency of negative behaviours in the workplace and the strategies used when exposed to these behaviours	Australia 74 nurses	Negative Acts Questionnaire—Revised; Purpose-designed questions; Ways of Coping questionnaire	Quantitative study	The most common type of negative workplace behaviour reported was “work-related bullying”, and they reported using a variety of coping strategies, including problem-focused strategies and seeking social support.
Hong et al. [35]	Investigate the effects of workplace bullying and different symptoms of post-traumatic stress and coping among hospital nurses	Korea 233 nurses	Workplace Bullying in Nursing—Type Inventory; Impact of Event Scale—Revised; Ways of Coping Checklist	Quantitative study	The study explores bullying in the workplace of nurses, detecting high-risk subgroups, and suggesting the development of coping interventions to reduce workplace bullying and symptoms of post-traumatic stress.
Mills et al. [36]	Determine whether nurses’ humour orientation styles and leadership styles can influence perceptions of workplace bullying	USA 459 participants	Multidimensional Sense of Humour Scale; Bass’s Multifactor Leadership Questionnaire; Negative Acts Questionnaire—Revised	Quantitative study	One of the four humour subscales, Humour Appreciation, affected perceptions of workplace bullying. The other three, Humour Recognition, Humour Production and Humour for Coping, had no effect. However, managers’ leadership styles affected reports of negative acts.
Peng et al. [37]	Determine the relationship between workplace bullying and nurses’ quality of life and also the mediating role of resilience between workplace bullying and quality of life	China 493 nurses	Negative Acts Questionnaire—Revised; 10-item Connor–Davidson Resilience; the Chinese Version of Professional Quality-of-Life Scale	Quantitative study	Bullying in the workplace had negative and direct effects on nurses’ quality of professional life. Resilience mediated the relationship between workplace bullying and quality of professional life.
Reknes et al. [38]	To investigate whether bullied nurses have a more negative coping style when faced with stressful events than nurses who are not bullied and to determine whether coping style moderates the relationship between bullying and anxiety	Norway 1st phase = 2059 participants 2nd phase = 1582 participants	Negative Acts Questionnaire—Revised; Hospital Anxiety and Depression Scale; Utrecht Coping List	Quantitative study	Bullied nurses use a goal-oriented active coping style less frequently than non-bullied nurses. Furthermore, active goal-oriented coping appears to be beneficial only when exposure to bullying behaviours is very low. Victims of bullying appear to deal more negatively with stressful events than others.
Tabakakis et al. [39]	Investigate the impact of workplace factors on nurses’ psychological resilience, including bullying	Australia 480 nurses	The Connor–Davidson Resilience 10 scale; The Practice Environment Scale of the Nursing Work Index; Negative Acts Questionnaire—Revised	Quantitative study	The work environment and perception of exposure to workplace bullying play a significant role in shaping nurses’ psychological resilience.
Yoo & Ahn [40]	Analyse the relationship between workplace bullying experiences, responses, and coping strategies	Korea 113 nurses	Workplace Bullying in Nursing-Type Inventory; Workplace Bullying in Nursing—Consequence Inventory; Way of Coping	Quantitative study	Nurses complained of helplessness, depression, stress, insomnia, and physical discomfort, and complained that they made more mistakes in their work and wanted to change jobs.

## Data Availability

The data are publicly available due to the nature of the review.

## Supplementary Materials

The following supporting information can be downloaded at: https://www.mdpi.com/article/10.3390/ijerph21040459/s1, File S1: Bullying against Healthcare Professionals and Coping Strategies: Scoping Review Protocol.
