# Supplementary material for: Bullying against Healthcare Professionals and Coping Strategies: A Scoping Review
Source: Int J Environ Res Public Health. 2024 Apr 9;21(4):459. doi: 10.3390/ijerph21040459 (PMC11050285; doi:10.3390/ijerph21040459)
Supplement: Supplementary file 1 [file ijerph-21-00459-s001.zip › ijerph-2890679-supplementary.pdf]

Bullying against Healthcare Professionals and Coping Strategies: Scoping Review Protocol

Complementary material

Scoping Review Protocol

**Research Question**			
Violence against healthcare professionals: Coping strategies in workplace bullying situations			
**Topics**			
Health professionals (AB)	Bullying (AB)	Coping strategy* (AB)	NAQ-R (AB)
Healthcare professionals (AB)	Mobbing (AB)	Coping strategy* (AB)	Negative Acts Questionnaire- Revised (AB)
Doctors (AB)	*civility (AB)	Cope (AB)	
Doctors (AB)		Coping (AB)	
Physicians (AB)		Coping skill* (AB)	
Nurses (AB)			
Nurses (AB)			
▼	▼	▼	▼
Combining with OR = #1	Combining with OR= #2	Combining with OR= #3	Combining with OR= #4
#1 AND #2 AND #3 OR #4			
**Database** (electronic databases only)			
EBSCO = 117<br>PubMed = 43<br>Web of Science = 116			
Restricted to: Full Text; peer reviewed; available in the library collection.<br>There was no limitation regarding the type of study design to be identified in the search.<br>The studies identified were restricted to English, Portuguese and Spanish.<br>The studies would have to have been published after 2010.			

**Inclusion criteria**
Studies that referred to violence against healthcare professionals in the workplace, that referred to the impact of this same violence and that mentioned coping strategies related to bullying in the workplace were included.
**Exclusion Criteria**
Studies that did not mention violence in the workplace and that did not mention violence against health professionals were excluded. Scoping review articles were also excluded.
